# Soil2Cover: Coverage path planning minimizing soil compaction for sustainable agriculture

**DOI:** 10.1007/s11119-025-10250-4

**Published:** 2025-06-03

**Authors:** Gonzalo Mier, Sergio Vélez, João Valente, Sytze de Bruin

**Affiliations:** 1 Laboratory of Geo-Information Science and Remote Sensing, Wageningen University & Research, Wageningen, 6708 PB The Netherlands; 2 JRU Drone Technology, Department of Architectural Constructions and I.C.T., University of Burgos, Burgos, 09001 Spain; 3 Centre for Automation and Robotics (CAR), Spanish National Research Council (CSIC), Madrid, 28500 Spain

**Keywords:** Controlled traffic farming, Soil compaction, Coverage path planning, Precision farming, Sustainable soil management

## Abstract

Soil compaction caused by heavy agricultural machinery poses a significant challenge to sustainable farming by degrading soil health, reducing crop productivity, and disrupting environmental dynamics. Field traffic optimization can help abate compaction, yet conventional algorithms have mostly focused on minimizing route length while overlooking soil compaction dynamics in their cost function. This study introduces Soil2Cover, an approach that combines controlled traffic farming principles with the SoilFlex model to minimize soil compaction by optimizing machinery paths. Soil2Cover prioritizes the frequency of machinery passes over specific areas, while integrating soil mechanical properties to quantify compaction impacts. Results from tests on 1000 fields demonstrate that our approach achieves a reduction in route length of up to 4-6% while reducing the soil compaction on headlands by up to 30% in both single-crop and intercropping scenarios. The optimized routes improve crop yields whilst reducing operational costs, lowering fuel consumption and decreasing the overall environmental footprint of agricultural production. The implementation code will be released with the third version of Fields2Cover, an open-source library for the coverage path planning problem in agricultural settings.

## Introduction

Soil compaction poses a major challenge to agricultural land management by degrading soil health and reducing crop productivity. Heavy machinery traffic is the main cause of both topsoil and subsoil compaction, affecting around 68 million hectares globally–half of which lies in Europe (FAO, 2015). Compaction increases soil density and reduces porosity, thereby impairing aeration, drainage, and root growth (Shah et al., 2017). It also disrupts plant growth by altering enzyme activity (Wang et al., 2019). Moderate compaction has been found to reduce crop yields by 5–40% (Nawaz et al., 2023; Van Orsouw et al., 2022). Given the multitude of impacts, minimizing soil compaction is crucial for sustainable farming and climate change mitigation (Machmuller et al., 2015).

Various techniques have been proposed to combat soil compaction. Deep tillage using chisel plows or subsoilers, and practices that improve soil organic matter can temporarily relieve compaction but often incur high costs and may further degrade soil structure (Shaheb et al., 2021). In contrast, preventive strategies that limit compaction before it occurs offer a more sustainable solution. A direct method to avoid compaction is to control the movement of heavy machinery. Controlled Traffic Farming (CTF) confines machine traffic to specific lanes, thereby protecting the bulk of the field from repeated passes (Gasso et al., 2013). This practice not only protects soil structure but also enhances crop productivity and sustainability. Successful CTF implementation requires optimized machine paths to minimize soil disturbance.

Coverage Path Planning (CPP) aims to generate routes that ensure complete field coverage with minimal overlap or missed areas (Ariza-Sentís et al., 2024). Recent research has introduced various CPP algorithms to address specific agricultural challenges. For instance, Juman et al. (2017) improved autonomous navigation in oil palm plantations using D-lite algorithms for real-time path planning to address labour shortages. Similarly, Jeon et al. (2024) developed a polygonal path planner for unmanned tillage in paddy fields, achieving a similar efficiency as manual operation. Bochtis et al. (2010) explored algorithmic solutions for in-field navigation to optimize paths for agricultural service units, improving efficiency and reducing compaction in large-scale machinery operations. Although these approaches have improved operational efficiency, most do not directly address soil compaction and overlook the cumulative impact of machinery passes (Chatzisavvas et al., 2023).

A few studies have integrated soil characteristics directly into path planning models. Bochtis et al. (2012) developed a decision support system (DSS) that uses electrical conductivity maps to assess soil sensitivity to compaction, optimizing machinery routes to reduce compaction risks. Similarly, Spekken et al. (2016) employed the RUSLE model to assess erosion, considering factors such as soil type and topography to optimize machine paths on steep terrains. However, these models do not explicitly account for the nonlinear nature of soil responses under repeated mechanical stress.

In contrast, the SoilFlex model (Keller et al., 2007) addresses this non-linear behaviour by simulating soil compaction and stress distribution using analytical stress propagation equations. It incorporates mechanical soil properties to predict displacement and rut depth, offering a comprehensive view on soil behavior under machinery loads.

In this paper, we describe Soil2Cover, an advanced coverage path planner that integrates the SoilFlex model into a novel soil compaction cost function. Our contributions are threefold:

- We design a cost function that captures the nonlinear behavior of soil compaction using SoilFlex, modeling the effect of repeated machinery passes.
- We develop a dual-graph route planning strategy that minimizes both route length and soil compaction, thereby reducing unnecessary re-tracing of field areas.
- We evaluate our approach on 1000 real-world fields under both single-crop and intercropping scenarios.

This paper quantifies the benefits of integrating the SoilFlex model by comparing routes avoiding soil compaction against minimal path length routes, where the latter conform to the most commonly used criterion in literature (Filip et al., 2020). The proposed approach supports sustainable farming practices-such as intercropping-and the use of autonomous agricultural machinery, thereby advancing precision farming and sustainable soil management practices.

## Materials and methods

### General overview

**Fig. 1:**
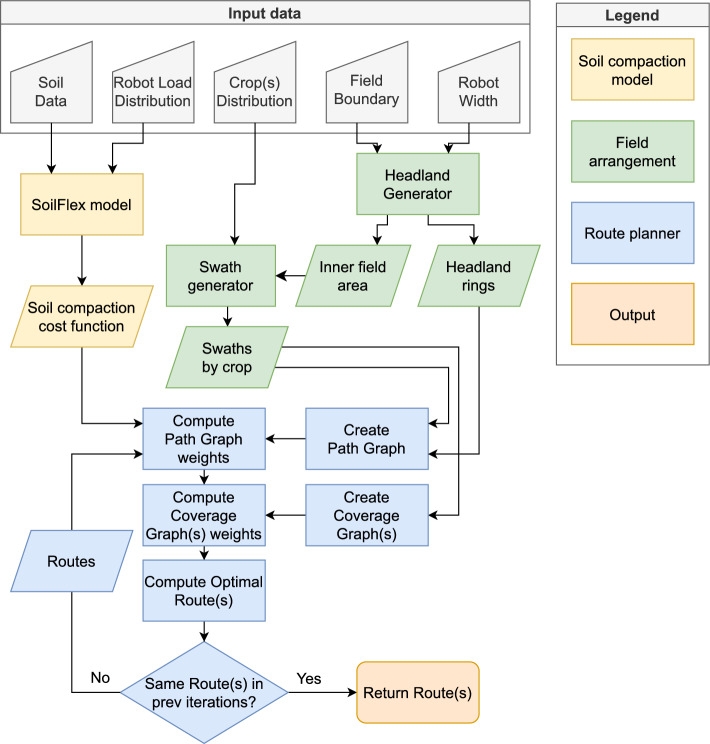
Flowchart of the Soil2Cover method. Block colours distinguish functionalities (see Legend)

The flow diagram of Fig. 1 shows the Soil2Cover method for solving an agricultural route planning problem while minimizing soil compaction. Required input data includes soil composition, robot load, crop distribution, field boundaries, and robot width. The information is processed by the SoilFlex model to develop a cost function that minimizes soil compaction and helps maintain soil health. A Headland Generator uses the field boundary and robot width to distinguish between the inner field area and the headland. Next, the Swath Generator creates swaths, accommodating different crop types in case of strip cropping or intercropping. Subsequent steps involve computing the weights for the path and coverage graphs that are essential for route planning. The coverage routing directly affects the soil compaction component of the cost function. An optimizer repeatedly assesses the cost function to compute the optimal route by continuously refining until the most efficient path for field coverage is determined. The loop terminates when subsequent iterations yield identical routes. Green and yellow blocks in the flowchart distinguish field area distribution from soil management, emphasizing the method’s integration with precision agriculture.

### Soil compaction under a single wheel

The path of agricultural vehicles directly affects soil disturbance. For a single wheel, a direct relationship can be established between the area of soil disturbed, and the path distance travelled over previously-undisturbed soil. Specifically, the disturbed area is approximately equal to the product of the width of the tyre and the path length (Mier et al., 2023b).

This relationship extends to soil compaction, stating that for a tyre producing constant normal stress along a straight path, the bulk density change is proportional to the path length and the bulk density change at a point within the disturbed area. This approximation is only valid if soil properties are homogeneous over the disturbed area.

To compute the bulk density change, this work employs the SoilFlex model (Keller et al., 2007). The SoilFlex model estimates soil stress and compaction by assessing the distribution of vertical stress beneath agricultural machinery. Under repetitive passes with the same load, the soil bulk density asymptotically approaches its saturation level using a logarithmic expression. The equations of this model are developed in Appendix 1.

For the scope of this research, the soil compaction produced by a single tyre following a path over homogeneous soil is approximated using the path length and the bulk density change below the tyre centre at 20 cm depth. This simplifying assumption is consistent with the SoilFlex model and our subsequent analyses and discussions.

### Soil compaction cost function

**Fig. 2:**
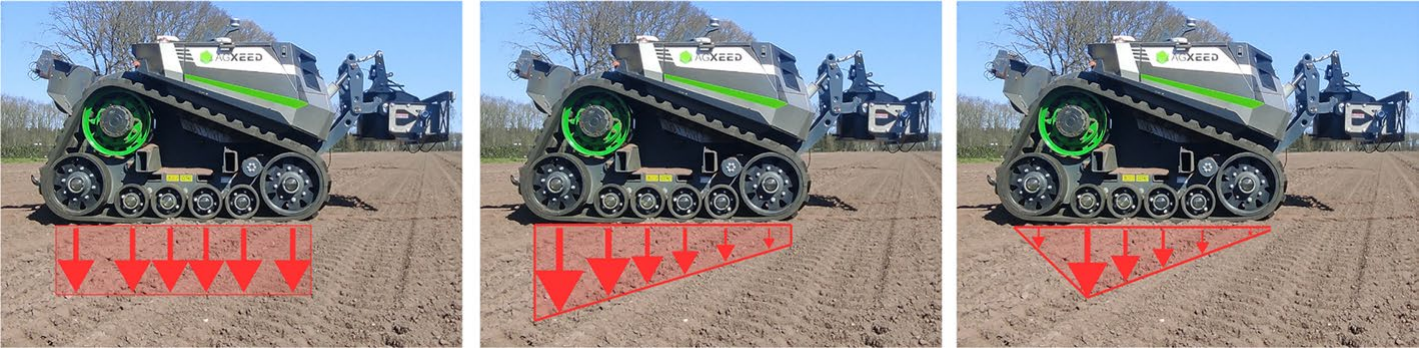
Load distribution on tracks according to Uniform, trapezoidal and triangular distributions

Tractors are typically characterized by a quad-tyre configuration or caterpillar tracks arranged in two lanes. Hence, the soil compaction affects twice the area compared to a single wheel lane. For brevity, this paper elaborates only on the case of caterpillar tracks. Particularly, the AgBot (Fig. 2) of the AgXeed company is used as an example. However, the methodology is equally applicable to any other type of wheeled or tracked vehicle.

**Table 1:** Relevant measures of the AgBot robot

Specification	Value
Total weight	7800 kg
Track length	2.55 m
Track width	0.61 m
Idlers per track	2
Idler radius	0.55 m
Rollers per track	4
Roller radius	0.3 m

Each AgBot track is composed of two idlers and four rollers (Table 1). The compaction made by a track on a traversed point is equal to the difference between the initial bulk density and the bulk density after idlers and rollers have passed. Note that the order in which each wheel stress is applied affects the final bulk density. An initial bulk density, $\rho _0$, is used to compute the bulk density after the first idler passed, $\rho _1$. Next, $\rho _1$ is the initial bulk density for computing the bulk density after the first roller passed, $\rho _2$. By the same reasoning, roller 2, 3 and 4 produce $\rho _3$, $\rho _4$ and $\rho _5$, respectively, and the second idler produces $\rho _6$. Consequently, the variation of bulk density made by the track is $\Delta \rho _{t} = \rho _6 - \rho _0$.

Let $T_\rho (\rho _{init})$ be a function that–given an initial bulk density $\rho _{init}$– returns the bulk density after the track passes. The function $B_\rho (n)$ is then defined to return the bulk density change made by a track after passing a point *n* times:

1 $$\begin{aligned} B_\rho (n) = {\left\{ \begin{array}{ll} B_\rho (n-1) + T_\rho (B_\rho (n-1)) \,\,\,\,\, \text {, if n }\ge 1 \\ T_\rho (\rho _{init}) \,\,\,\,\, \text {, otherwise} \\ \end{array}\right. } \end{aligned}$$

Even when the load of the robot is evenly distributed across two tracks, the load distribution within each track (Fig. 2) may be uneven. Uniform load distribution weights for each wheel in a track were compared against trapezoidal (Wong et al., 2019) and triangular (Keller & Arvidsson, 2016) load distributions of weight, using the AgBot data listed in Table 2.

**Table 2:** Load distribution values for each wheel in Agbot tracks. Each value is the fraction of load applied to that wheel

Load distribution type	Idler 1	Roller 1	Roller 2	Roller 3	Roller 4	Idler 2
Uniform	0.167	0.167	0.167	0.167	0.167	0.167
Trapezoidal	0.016	0.086	0.139	0.192	0.245	0.319
Triangular	0.050	0.102	0.184	0.265	0.347	0.050

**Fig. 3:**
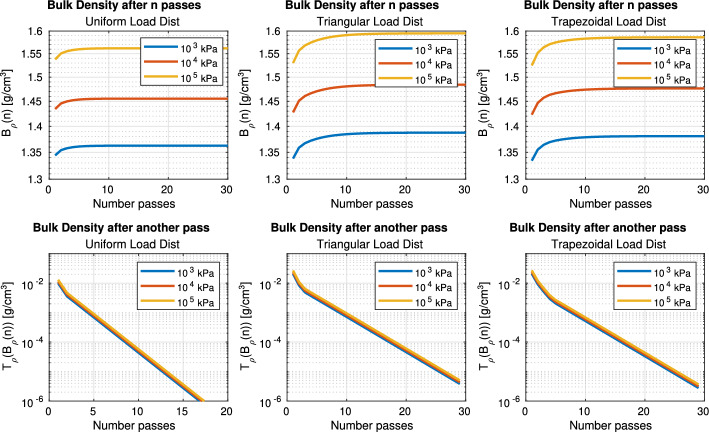
SoilFlex-computed bulk density after *n* passes with the robot. The number of passes is represented by the x-axis. The columns refer to results using Uniform, Triangular and Trapezoidal load distributions, respectively

Figure 3 shows the comparative analysis of how the three load distributions modify the bulk density values obtained, starting with a bulk density of $B_\rho (0) = [1 g/cm^3]$. Each load distribution is represented in a row. Each line in a plot represents a different total load applied. In the columns, the plot shows the evaluated values of $B_\rho (n)$ and $T_\rho (B_\rho (n))$, respectively. Note that the y-axis in the second-row plots has logarithm scale.

Even though an increase in stress produces greater soil compaction, the ratio between the results from different loads is constant. Therefore, for a field with a soil with homogeneous properties and assuming that the robot has constant weight, the weight of the robot has no impact on the route that minimizes the soil compaction.

However, the load distribution model does produce different soil compaction results. In contrast to the uniform load distribution, the triangular and trapezoidal load distributions yield similar results. Since an uniform load distribution is unrealistic, this study adopts a trapezoidal load distribution.

For each route segment, the soil compaction cost is computed as:

2 $$\begin{aligned} f_c(n) = \Omega _L * B_\rho (n) \end{aligned}$$

where $\Omega _L$ is the effective travel length of the route segment, and $B_\rho (n)$ is the increase in soil bulk density after *n* passes, i.e, it models the compaction resulting from repeated machinery passes. Since the track width is constant, $\Omega _L$ implicitly quantifies the area compacted by the machine passes. The total soil compaction cost produced by a complete route is the sum of the costs of all the route segments.

### Headland generation

For several operations, revisiting a previously covered swath would damage the crop. Therefore, manoeuvres between swaths occur in the headlands. A headland is an area reserved to make turns from swath to swath or to travel around the field without damaging the crop in the inner field.

Following common practice (Nilsson & Zhou, 2020), the headland is here generated by inward buffering the borders of the field (including obstacles) by three times the width of the robot. Moreover, a linear ring between the inner and outer borders of each headland is created. In this work, this line is called a headland ring, and is denoted by $H^i_{p=j}$, where $i \in [1, I]$ is the index of the headland, with 1 referring to the outer border, and I to the inner border, and $j \in [1, J_i]$ the $j^{th}$ point in the $i^{th}$ headland ring, with $J_i$ being the number of points in the headland ring *i*.

### Swaths planner

Swaths are used by the robot to cover the field while traversing the inner field during the operation. Swaths are generated by intersecting parallel straight lines with the area of the inner field, until the entire field is covered. The distance between consecutive lines equals the width of a crop strip. The orientation of the swaths can be predetermined, for example in the case of orchards or already cultivated land, or it can be optimized according to an objective function (Mier et al., 2023a). The objective function used in Soil2Cover for planning the swaths in the inner field is the sum of the lengths of the swaths. This is consistent with the fact that for a single pass the soil compaction is proportional to the length of the route. Swath angle optimization uses an exhaustive search with a step size of $\pi /180$ (Mier et al., 2023a).

### Single crop

In this section, the route planner for a single crop is explained following the pseudocode presented in Algorithm 1. The initialization steps ($\#$1) in this algorithm refer to the Sections 2.3, 2.4 and 2.5, respectively.

**Algorithm 1:**
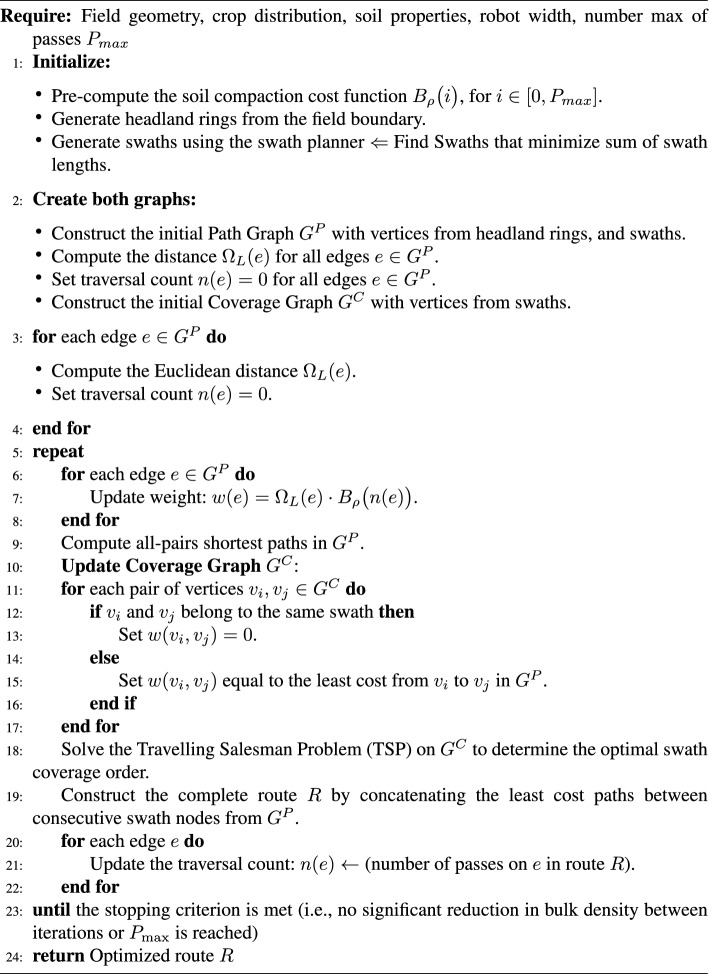
Soil2Cover Route Optimization

#### Graph definition

A graph, defined by $G = (V, E)$, is a structure that contains a set of nodes, called vertices "*V*", and the relationship between pairs of vertices, called edges "*E*". Each edge has an associated weight representing the cost to traverse it. If two vertices are not connected, the weight between them is $+\infty$.

In Soil2Cover, a 2-bidirectional-graphs approach is used to solve the route planning problem in agriculture. Those graphs are the Path Graph ($G^P$) and the Coverage Graph ($G^C$).

#### Path Graph

**Fig. 4:**
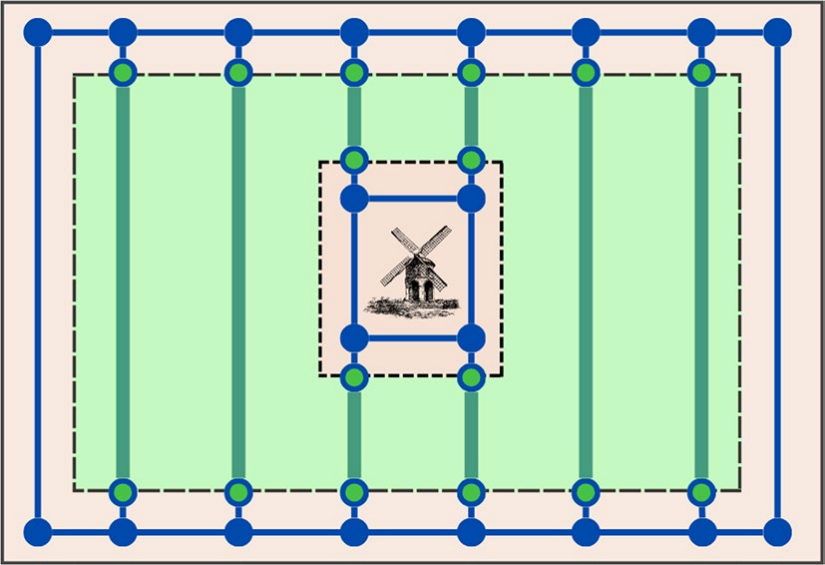
Path Graph example. Nodes and edges of the Path Graph are shown in blue. Green points with a blue border are part of both the Path Graph and the Coverage Graph. Swaths are represented by green lines; these and are not edges of the Path Graph. The headlands are shown in light brown; the inner field is green

The Path Graph, $G^P = (V^P, E^P)$, (Fig. 4) is used to find the shortest path to travel through the headlands between swaths. Vertices of $G^P$ are points, and edges are line segments. The Path Graph is populated with nodes and edges in three steps.

First, headland rings points ($H^i_{p=j}$) are added to $V^P$, and the connection between consecutive points ($H^i_{p=j} \leftrightarrow H^i_{p=j+1}$) in the same headland ring are added to $E^P$.

Second, for each swath, and for each point on it ($s_p$), the closest point on the headland rings is found ($s'_p$). $s_p$ and $s'_p$ are added to $V^P$ and the connection between $s_p$ and $s'_p$ is added to $E^P$. This procedure also applies to the start and end points of the route.

Third, redundant edges are removed. Since the soil compaction cost function requires knowing how many times an edge has been traversed, every pair of overlapping edges is transformed into non-overlapping segments. Then, for each node $v^P_j \in V^P$ and edge $e^P_{ik} \in E^P$, with $i \ne j \ne k$, where $v^P_j$ is the $j^{th}$ vertex and $e^P_{ik}$ is the edge connecting vertices *i* and *k*. For each pair $v^P_j$ and $e^P_{ik}$, if the point corresponding to $v^P_j$ is located on the segment that joins the points $v^P_i$ and $v^P_k$, the edge $e^P_{ik}$ is removed from $E^P$, and the edges $e_{ij}$ and $e_{jk}$ are added to $E^P$. We consider that $v^P_j$ is on the segment $(v^P_i,\, v^P_k)$ if the distance from the point to the segment is less than $\delta _{tol}$.

The weight of an edge $e^P_{ij} \in E^P$ is the Euclidean distance between $v^P_i$ and $v^P_j$.

#### Coverage Graph

**Fig. 5:**
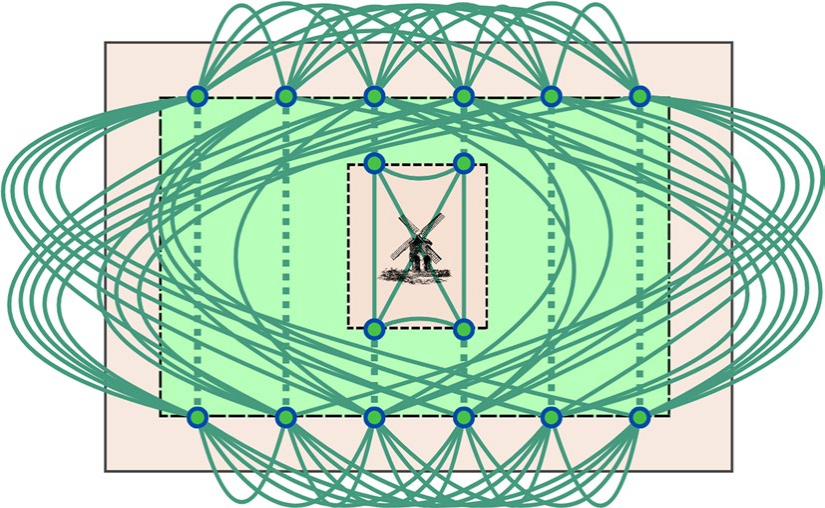
Coverage Graph represented over a field. Green lines are connections between nodes, dotted green lines are edges with 0 cost, connecting nodes on the same swath. Every pair of nodes on the same headland is connected

The Coverage Graph, $G^C = (V^C, E^C)$ (Fig. 5), is used to define the space of possible routes.

Each swath is represented by its two endpoints. A swath is considered visited after both endpoints are visited, with their order determining the traversal direction. $V^C$ contains both endpoints of every swath and the field entry point. In addition, an important property is that $V^C \subset V^P$, meaning the nodes on the Coverage Graph are also represented in the Path Graph (Bochtis & Vougioukas, 2008).

The weight of the edge $e^C_{ij} \in E^C$, being $v^C_i$ and $v^C_j$ points of the same swath is 0. Otherwise, the weight is the cost of the shortest path from $v^C_i$ to $v^C_j$ in the $G^P$. The shortest path between each pair of vertices in $G^P$ is computed using the Floyd-Warshall algorithm (Floyd, 1962). The Floyd-Warshall algorithm efficiently computes the shortest paths between all pairs of vertices in a weighted graph. If two vertices are not connected in $G^P$, they are neither connected in $G^C$.

#### Finding the shortest route

The Coverage Graph problem is equivalent to the Travelling Salesman Problem (TSP). The TSP is the problem of finding, for a given graph, the shortest Hamiltonian path, which is a path that visits all the vertices of the graph. This problem is NP-hard, as there is no known method to find a solution in polynomial time. Fortunately, there are open-source solutions like Or-tools (Perron, 2011), which provide optimizers to find near-optimal solutions in reasonable time.

Once the optimizer returns the coverage order of the swaths, the transition path between swaths is searched in the $G^P$.

### Minimizing soil compaction for single crop

To find a route that minimizes soil compaction, Soil2Cover uses an iterative algorithm. First, the function $B_\rho (n)$, previously defined, returns the difference of bulk density between the initial bulk density and its value after the $n^{th}$ pass (Fig. 3). Next, $G^P$ and $G^C$ are created following the same procedure as in Section 2.6. The weights of $G^P$ are replaced by the cost of passing through each edge one more time. Equivalently, the cost of the edge $e_{ij}$ equals $d_{ij} * B_\rho (n_{ij})$, being $n_{ij}$ the number of passes between vertices *i* and *j*, and $d_{ij}$ is the Euclidean distance between vertex *i* and *j*. In the first iteration, the costs of $G^P$ to minimize soil compaction are proportional to the costs of $G^P$ to minimize distance.

$G^P$ is used to compute the weights of $G^C$, then find an initial coverage route, and generate the complete route. Once the coverage route is generated, it is split between segments that correspond to edges in $E^P$. For each edge $e^P_{ij}$, $n_{ij}$ is updated with the number of times that segment is traversed in the route, regardless of its direction. The latter numbers are used to update the weights of $G^P$. $G^P$ is again used to generate the new weights of $G^C$ and to produce a new route.

This repeats until the obtained route remains unchanged for two consecutive cycles. The method returns the route that has minimized the total soil compaction.

### Minimizing soil compaction with strip cropping

In the case where multiple row crops are planted in alternating strips, *M* refers to the number of crops in the field. Each crop has its own swaths $S_i$, where $1 \le i \le M$, covered either by different robots or by the same robot at different times. For this case, Soil2Cover has a common Path Graph, $G^P$, for all crops, while creating a unique Coverage Graph, $G^C_i$, for each crop ($1 \le i \le M$). Note that generating $G^P$ with points from all swaths (regardless of crop) ensures $V^C_i \subset V^P$ for all crops *i*. The algorithm followed is the same as for a single crop, with the difference being that the route for all crops is generated before updating the weights of $G^P$. Consequently, $n_{ij}$ is the number of times an edge $e_{ij}$ in $E^P$ has been traversed, regardless of which robot visited the edge. The rest of the steps and the stopping condition remain similar.

### Experiments

To select a representative set of fields, we retrieved boundary polygons from the https://github.com/Courseplay/CourseGenerator repository in.xml format. Each file in this repository contains multiple fields. We retained only fields with an area between 1 and 20 ha and exported them to.wkt (well-known text) format, yielding 1567 fields. We retained the first 1000 fields to limit computation time while still capturing a broad range of shapes and sizes for our experiments.

Both single crop with 3 m row width (AgBot width) and strip cropping with two crops in 6 m width as in Campanelli et al. (2023) were considered. In both cases, the robot operation concerned one, two and three passes over the crop cycle. As soil compaction reduces by reusing previous paths, robots operations for the same crop were constrained to follow the same route. To include this case in Soil2Cover, $f_{c}(n_{ij})$ is replaced by $\sum _{k=1}^2 f_{c}(2*n_{ij} + k)$ or $\sum _{k=1}^3 f_{c}(3*n_{ij} + k)$, for simulating repeating the operation two and three times respectively.

All analyses and graphics were produced using C++, GDAL (GDAL, OGR contributors, 2022) and Or-tools (Perron, 2011), and with Matlab 2023b. The laptop used for experiments was an MSI GF627RE with an Intel(R) Core(TM) i7-7700HQ CPU, running Ubuntu 22.04.5.

Experiments were done using the Fields2Cover library, a software tool specifically designed to optimize coverage path planning (CPP) for agricultural machinery. It allows considering various factors like soil properties, vehicle characteristics, and environmental constraints to implement path planning algorithms (Mier et al., 2023a).

## Results

### Single crop

**Fig. 6:**
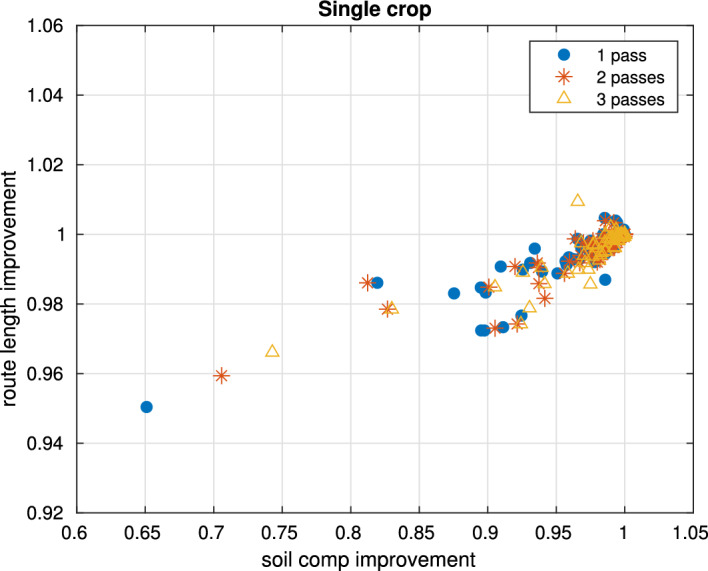
Ratios of soil compaction and route length, between routes obtained optimizing soil compaction and routes optimizing route length. Routes account for 1, 2 and 3 passes, for a single crop at 3 m width

Figure 6 shows the ratios of soil compaction and route length between different routes. Let $R_A$ be the baseline route obtained by optimizing solely for route length, with soil compaction $S_A$ and route length $L_A$. For any alternative route $R_B$, with soil compaction $S_B$ and route length $L_B$, its ratio is defined as $(S_B/S_A,\, L_B/L_A)$. Thus, a ratio of $(1,\,1)$ implies that $R_B$ has identical soil compaction and route length to the baseline route $R_A$. A value below 1 for either component indicates an improvement (i.e., lower soil compaction or shorter route) relative to the baseline.

After optimizing the route for 1000 fields with a single pass, 66 of them produced different solutions depending on the cost function. Unexpectedly, for single crops, optimizing for soil compaction produced better results in both soil compaction and route length compared to route length optimization. This likely occurred because modifying the weights in $G^C$ helped the optimizer escape local minima. The average soil compaction improvement for the 66 fields with one, two, and three passes were [0.964, 0.971, 0.978], while route length improvement were [0.994, 0.995, 0.996].

**Fig. 7:**
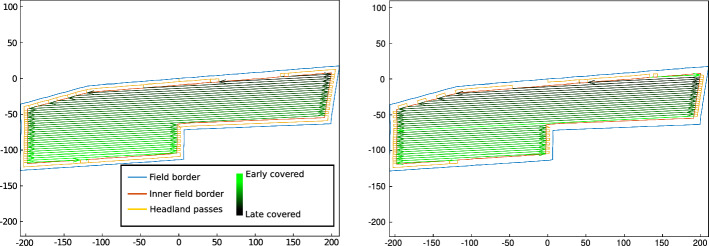
Optimized routes for the same field according to two objectives. Left: minimal length route. Right: minimal soil compaction route. The coordinates in both sub-figures are in meters relative to the start and end point of the route

Figure 7 shows the obtained route for a field optimized for route length and for soil compaction. On this field, when the route minimizes soil compaction, the bottom right headlands are not travelled, to reduce the damage to the soil in that area.

### Strip cropping of two crops in 6 m wide strips

**Fig. 8:**
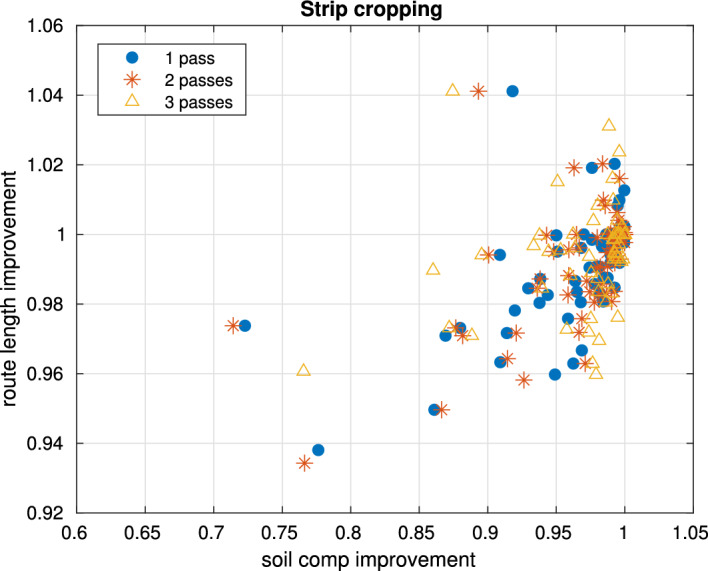
Ratios of soil compaction and route length, between routes obtained optimizing soil compaction and routes optimizing route length. Routes account for 1, 2 and 3 passes, for two crops at 6 m width

Figure 8 shows the route improvement ratios for a strip cropping scenario with two crops grown in alternating 6 m wide strips. For the one-pass case, 66 out of the 1000 fields yielded a different route than the baseline (i.e., the route obtained when minimizing path length), while for the three-pass case, 130 fields were assigned a different route. For the former 66 fields, we computed the average ratios of the soil compaction and route length values (with the ratios defined as the metric for the soil compaction optimized route divided by that for the baseline route, $(S_B/S_A,\, L_B/L_A)$). The average soil compaction ratios for one, two, and three passes were [0.964, 0.966, 0.973], respectively, while the corresponding average route length ratios were [0.991, 0.992, 0.995]. Recall that ratios below 1 indicate improvements compared to the baseline.

**Fig. 9:**
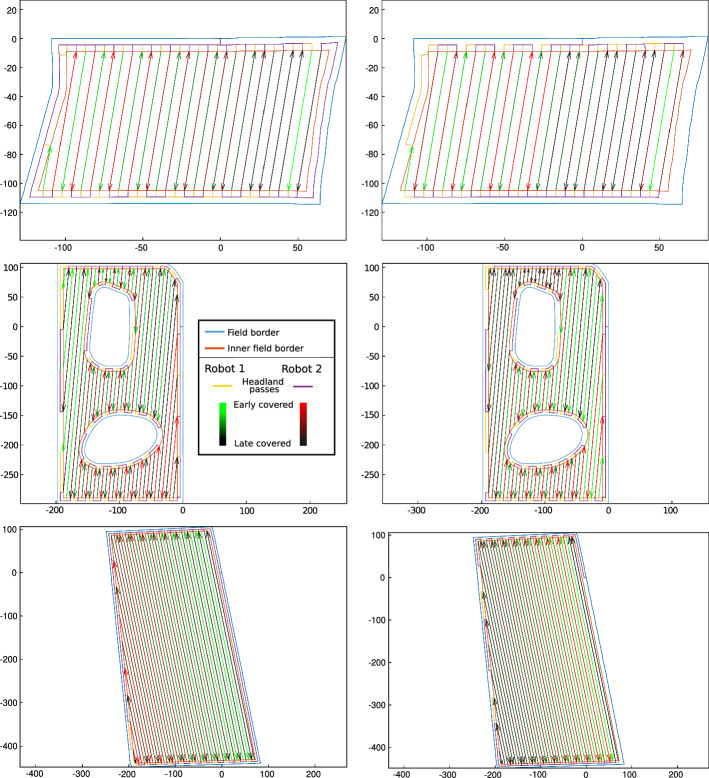
Optimized routes on three fields (rows) according to two cost functions (route length, left; soil compaction, right). In these scenarios, two robots operate in the same field, doing strip cropping. Swaths are assigned alternately: odd-numbered swaths to one robot, even-numbered swaths to the other. Both robots start at the origin of the local coordinate system. Axes are in meters

Figure 9 shows the routes through three fields, for the two considered cost functions. The first case concerns a small rectangular field; To minimize the soil compaction, the optimizer produces a path without lateral headland passes. The second field contains two large obstacles. In this case, the swath coverage order changes significantly: the first robot begins in the northwest corner to minimize route length, but in the southeast corner to minimize soil compaction. In the last field, Soil2Cover employs the same strategy as in the first, avoiding the right edge to prevent additional soil compaction.

## Discussion

We have demonstrated that routing autonomous tractors along fixed paths can reduce soil compaction risk, thereby preserving soil health and boosting crop yields (Fig. 6). Our approach reduces deterioration of soil structure and improves efficiency by reducing operational route length. Predictable traffic routes integrate seamlessly with precision farming systems, enhancing input efficiency and supporting sustainable management. Although farmers recognize the need for Controlled Traffic Farming (CTF) practices that limit autonomous tractors circulation to permanent tracks (McPhee et al., 2020), operational challenges often hinder their adoption (Tamirat et al., 2022). In this context, an autonomous route planning algorithm that adheres to CTF principles represents a key means for preserving soil health.

### Novelty of the work

The integrated SoilFlex model (Keller et al., 2007) treats soil compaction as a complex, nonlinear process. By accounting for soil properties, equipment weight, and pass frequency, Soil2Cover offers a meticulous representation of field conditions and a deeper understanding of machinery’s impact on soil. Unlike previous methods (Plessen, 2018; Bochtis et al., 2012; Spekken et al., 2016), it minimizes soil damage by evaluating changes in bulk density from vehicle passes-focusing on the disproportionate impact of initial passes on surface layers (Patel & Mani, 2011; Pulido-Moncada et al., 2019). Simulations on 1000 fields using a single 3-meter machine, Soil2Cover improved route efficiency by up to 4–6% and reduced compaction by up to 30% (Fig. 6). Even without explicit compaction optimization, the algorithm consistently found efficient routes that reduce route length and environmental impact.

The algorithm also handles complex field shapes and obstacles, ensuring efficient land coverage while protecting soil quality–a critical requirement for sustainable agriculture. Its two-graph method, using the Floyd-Warshall algorithm, outperforms distance-only approaches by directly minimizing soil damage. Moreover, by integrating the SoilFlex model, Soil2Cover treats soil as a heterogeneous continuous system and offers route planners a new strategy to mitigate soil compaction and preserve soil structure.

### Intercropping and strip cropping

Soil2Cover also supports routes for intercropping. It customizes routes by accounting for each crop’s machinery and compaction sensitivity. While intercropping (or mixed cropping) and strip cropping are known to boost productivity and preserve soil fertility (Brooker et al., 2015; Hernández-Ochoa et al., 2022), these practices demand tailored machinery to meet each crop’s unique requirements. For example, perennial plants, such as fruit trees, require distinct management compared to annual crops (Hauggaard-Nielsen et al., 2012; Ma et al., 2007; Martin-Gorriz et al., 2022; Wei et al., 2024).

In our intercropping simulation-planting crops in 6 m strips (Campanelli et al., 2023)-soil compaction improvements matched those in single-machine trials, with path lengths varying by up to 6% and compaction dropping by up to 30% (Fig. 6). These results further validate Soil2Cover’s capacity to enhance machinery use while reducing operational time, fuel consumption, and environmental impact.

Additionally, the technique can integrate with low-pressure tyres, advanced tracks, and gantry conveyors to further reduce compaction (Mileusnić et al., 2022). Adjusting SoilFlex inputs enables more optimized paths. The algorithm’s flexibility also permits integration with multi-robot soil mapping (Roberts-Elliott et al., 2022) and real-time wheel track identification (Zhang, 2024), enabling dynamic path adaptation based on fresh field data. Future versions may consider diverse agricultural vehicles, as some operations permit tyres with lower inflation pressures to expand the tyre-soil contact area and cut compaction (Shaheb et al., 2021). In this work, the approach was tested on straight swaths. The method is expected to work on curved swaths as well, and this will be tested in future research.

## Conclusions

Soil2Cover provides a key tool for sustainable soil management. By integrating the SoilFlex model and targeting machinery pass frequency rather than merely tyre load, it offers a precise strategy to reduce soil compaction–a major threat to productivity and soil health.

Simulations on 1000 fields showed that Soil2Cover improved route efficiency by 4–6% in 6–13% of the fields and reduced compaction by up to 30% in both single-machine and intercropping scenarios. Its robust performance in complex fields demonstrates its capacity to protect soil while guiding robotic tractors with high precision. Notably, its cost function captures the nonlinear dynamics of soil compaction and the outsized impact of initial passes, which is vital for planning routes that prioritize soil health over simple distance minimization. Moreover, by customizing routes for intercropping, Soil2Cover optimizes yield while safeguarding soil health.

Future work will test Soil2Cover across diverse field conditions and soil types to confirm its robustness. A next step would be to relax the assumption of homogeneous soil properties across the entire field. This advancement can be combined with real-time in-field sensing of soil and weather conditions to further optimize operational timing, reduce compaction, cut costs, and increase yields.

## Appendix 1

The type of tractor wheel affects the pressure applied to the ground. In the case of wheels, SoilFlex (Keller et al., 2007) describes the vertical stress as:

3 $$\begin{aligned} \sigma (y) =&C_A*\left( \frac{w(x)}{2} - y\right) *e^{-\gamma *\left( (w(x)/2) - y\right) }&\text {, if }|y|\le \frac{w(x)}{2}\end{aligned}$$

4 $$\begin{aligned} \sigma (x) =&\sigma _{x=0,y}*\left( 1-\left( \frac{x}{l(y)/2}\right) ^\alpha \right)&\text {, if }|x|\le \frac{l(y)}{2} \end{aligned}$$

while the vertical stress for caterpillar tracks are defined by Keller and Arvidsson (2016) as:

5 $$\begin{aligned} \sigma (y) =&\sigma _{max} * \left( 1 + \frac{|y| - W/2}{W/2}*(1-a)\right) / a&\text {, if }|y|\le \frac{W}{2} \end{aligned}$$

6 $$\begin{aligned} \sigma (x) =&\sigma _{x=0,y} * \cos {\left( \frac{2 \pi x}{L}\right) }/2&\text {, if }|x|\le \frac{L}{2} \end{aligned}$$

where $C_A$, $\gamma$, $\alpha$ and *a* are tyre parameters (Keller et al., 2007; Keller & Arvidsson, 2016), $\sigma _{x=0,y}$ is the stress under the centre of the tyre, *l*(*y*) and *L* are the length of the tyre, and *w*(*x*) and *W* are the width of the tyre.

Vertical stress is distributed over the contact area, $A_{contact}$, discretized into *i* elements with $A_i$ area and $\sigma _i$ normal stress (Söhne, 1953). The radial normal stress, $\sigma _{r,i}$ at depth z, assuming negligible horizontal stress, is

7 $$\begin{aligned} \sigma _{r,i} = \frac{\xi \sigma _i A_i}{2 \pi r_i^2} \cos ^{\xi -2}{\theta _i} \end{aligned}$$

where $\xi$ is concentration factor, $r_i$ is the distance from the centre of $A_i$ to the desired point, and $\theta _i$ is the angle between the normal load vector and the vector from the centre of $A_i$ to the desired point (Keller et al., 2007).

Using the principal stresses and the first stress invariant (Koolen & Kuipers, 1983), the mean normal stress, p, is:

8 $$\begin{aligned} p = \frac{1}{3} \sum _{i=0}^n \sigma _{r,i} \end{aligned}$$

SoilFlex employs O’Sullivan and Robertson (1996) to model rebound and recompression effects of the soil, based on the virgin compression line (VCL), recompression line (RCL), and steeper recompression line (RCL’).

9 $$\begin{aligned} VCL:&\upsilon = N - \lambda _n \ln p \end{aligned}$$

10 $$\begin{aligned} RCL:&\upsilon = \upsilon _{init} - \kappa \ln p \end{aligned}$$

11 $$\begin{aligned} RCL':&\upsilon = \upsilon _{YL} - \sqrt{\lambda _n \kappa } \ln p \end{aligned}$$

where $\upsilon$ is the specific volume, $\upsilon _{init}$ is the initial specific volume and $\upsilon _{YL}$ is the volume at the intersection of the yield line and the recompression line. *N* is the specific volume at $p = 1kPa$, $\lambda _n$ is the compression index and $\kappa$ is the recompression index (Keller et al., 2007). Finally, the Bulk Density, $\rho$, is computed as

12 $$\begin{aligned} \rho = \frac{\rho _s}{\upsilon } \end{aligned}$$

where $\rho _s$ is the density of solids.

In SoilFlex, parameters such as the compression index $\lambda _n$, the recompression index $\kappa$, the initial specific volume $\upsilon _{init}$, and the load distribution factors ($C_A$, $\gamma$, $\alpha$, etc.) are used to compute bulk density changes. Greater values for compression or recompression indices increase predicted soil deformation under load. Conversely, parameter sets indicating lower soil susceptibility (e.g., smaller $\lambda _n$) yield smaller incremental bulk density changes, making certain routes more favorable. Thus, adjusting these model parameters to represent the local soil type and machinery characteristics directly influences route selection, allowing Soil2Cover to prioritize or avoid certain zones based on their predicted compaction risk.
